# Adolescent-Selected Food Intake and Engagement with the External Food Environment Contribute to Unhealthy Diets in Accra, Ghana

**DOI:** 10.1016/j.cdnut.2026.109435

**Published:** 2026-07-10

**Authors:** Elodie Becquey, Rock R Zagré, Deda Ogum, Lieven Huybregts, Richmond Aryeetey, Marie T Ruel, Jef L Leroy

**Affiliations:** 1 Nutrition, Diets, and Health Unit, International Food Policy Research Institute, Washington, DC, United States; 2 Nutrition, Diets, and Health Unit, International Food Policy Research Institute, Dakar, Senegal; 3 Department of Population, Family & Reproductive Health, University of Ghana School of Public Health, Accra, Ghana

**Keywords:** adolescents, food selection, diet quality, food environment, food policy, Ghana, nutrient adequacy, urban

## Abstract

**Background:**

As adolescents gain autonomy, they interact more independently with their food environments, increasing their vulnerability to rapidly changing and often unhealthy food environments. In Accra, Ghana, unhealthy foods and beverages are widely available and heavily marketed.

**Objectives:**

This study aimed to assess the healthiness and nutrient adequacy of adolescents’ diets in Accra and to examine whether diet healthiness is associated with independent adolescent food selection and their engagement with home, school, and outlet food environments.

**Methods:**

We conducted a cross-sectional study using quantitative 24-h dietary recalls among 911 adolescents aged 12 to 18 y living in 5 low- and 5 middle-class neighborhoods in Accra. For each food and beverage consumed, we documented who selected it and where it was obtained and consumed. We calculated the global diet quality score (GDQS) and the prevalence of adequate intake for fruits and vegetables, protein, fiber, total sugar, total fat, saturated fat, and 11 micronutrients. Mixed-effects models assessed associations between independent adolescent food selection and food acquisition location (home, school, or other food outlets), and diet healthiness and macronutrient and micronutrient intake.

**Results:**

A large proportion of adolescents consumed excessive amounts of total sugar (49%) and total fat (44%) and inadequate amounts of fruits and vegetables (98%), fiber (45%), and 8 of the 11 micronutrients assessed (39%‒100%). Greater adolescent-selected food intake (mean, 62% of energy intake) was associated with lower GDQS and intakes of fruit and vegetables, protein, fiber, and 6 micronutrients and with higher sugar intake (*P* < 0.05). Greater reliance on foods from the external food environment (school: 14%; other outlets: 46%) showed similar associations with poorer dietary indicators.

**Conclusions:**

Strengthening the design, implementation, and enforcement of coordinated food policy bundles is needed to shift adolescents’ preferences toward healthy foods and reduce the availability, accessibility, and appeal of unhealthy options in their food environments.

## Introduction

Adolescence is a period of increasing independence during which young people start engaging directly with their food environment and making their own decisions about what to eat. The Innocenti framework on food systems for children and adolescents highlights the dynamic interplay between adolescents’ growing autonomy, the food environments they encounter, and the broader food system drivers that shape their diets and nutrition [1]. As adolescents gain agency and interact with increasingly diverse—and often unhealthy—food environments, their dietary behaviors and nutritional status are influenced in ways that have important implications for their diet and health, both in the short and long term [2]. Adolescents also have high micronutrient requirements because of rapid growth [3], increasing their vulnerability to poor-quality diets. Evidence on how adolescents select their food and on the specific food environment factors influencing this selection is essential to guide policies that support healthy diets during adolescence and into adulthood.

Unhealthy diets are a major public health concern in Sub-Saharan Africa, which faces a double burden of malnutrition—defined as the coexistence of undernutrition alongside overweight, obesity, and diet-related noncommunicable diseases (NCDs) [4,5]. Ghana, a lower-middle-income country in West Africa, is an example of a country affected by the double burden of malnutrition [6], with one of the highest rates of overweight (25%) and obesity (15%) in Sub-Saharan Africa [5] coexisting with high prevalence of anemia (41%) among females of reproductive age [7]. In the Greater Accra region, about one-third of females of reproductive age and 9% of males were affected by obesity in 2022, whereas <5% of adults were too thin for height, defined by a BMI (in kg/m^2^) of <18.5 [7]. In contrast, among adolescents aged 15 to 19 y, 12% of girls and 39% of boys were too thin for height, defined by a BMI-for-age lower than −1 SD, whereas obesity was less prevalent (7.1% and 2.7%, respectively) [7].

Although adolescent obesity is still relatively low in Accra, adolescence represents a critical window for establishing lifelong healthy dietary behaviors and preventing overweight, obesity, and NCDs [8]. In Ghana, several healthy food environment policies to address the multiple burdens of malnutrition are under development, and among them, the regulation of unhealthy food promotion to children was considered the highest priority by experts [9]. However, detailed data on adolescents’ diets and their drivers remain limited, even though such information is essential for effective policy design, implementation, and targeting. Emerging evidence shows that the food environment in Greater Accra is characterized by the widespread availability, affordability, and marketing of unhealthy foods and beverages, in and around schools [[10], [11], [12]]. A qualitative study from that region found that adolescent food choice at school was influenced by multiple factors, including digital and in-person marketing, perceived hygiene and food quality, affordability, and interactions with food vendors [13]. Peer perceptions and parental guidance also shaped food choice. However, little quantitative evidence exists on the extent to which adolescents’ diets are shaped by their engagement with the food environment, their decision making on food selection, or both. Furthermore, evidence remain limited regarding related gaps in nutrient adequacy among adolescents in this setting [14].

The first objective of our study was to describe the healthiness and nutrient adequacy of adolescents’ diets in low- and middle-class neighborhoods in Greater Accra, Ghana. Building on this description and highlighting the main gaps in healthiness and nutrient intake, the second objective was to assess whether variation in the healthiness and nutrient intake of adolescents’ diets was associated with adolescent food selection and the degree to which they engaged with the external food environment.

## Methods

### Study area

The study was conducted in Accra, the capital city of Ghana. Ten neighborhoods representing low-class (*n* = 5) and middle-class (*n* = 5) living conditions were selected. We started with an existing list of 100 neighborhoods created by grouping Ghana Statistical Service enumeration areas into neighborhoods, as perceived by inhabitants and delimited by natural barriers [15]. We used principal component analysis to create a housing quality index using data from the most recent census on access to electricity, source of water, type of toilet, type of bathing facility, methods of waste disposal, cooking fuel, kitchen facility, and number of persons per sleeping room [15,16]. We ranked the neighborhoods based on this index and excluded the top quintile (neighborhoods with the best housing quality and the fewest substandard housing characteristics) to restrict our sample to low- and middle-class neighborhoods (Supplemental Figure 1). Eleven neighborhoods with fewer than 500 households were also excluded because they were too small to meet the cluster-specific target sample (see below). Five neighborhoods with >10,000 households were excluded because of the high cost of conducting the household and food outlet census in neighborhoods that large. The remaining neighborhoods were classified into housing quality index tertiles. In total, 5 neighborhoods were randomly selected from the lowest tertile (the stratum representing low-class neighborhoods) and 5 from the highest tertile (the stratum representing middle-class neighborhoods; Supplemental Figure 1).

### Study design and study participants

The study used a cross-sectional design. Study participants were adolescents aged 12 to 19 y who lived in one of the study neighborhoods and were enrolled in a school located in, or within 1 km of, the study neighborhood’s perimeter. We excluded adolescents aged 10 to 12 y because the 24-h dietary recall method may not accurately reflect food intake in this younger age group [17]. Adolescents with a health or cognitive condition requiring a special diet or preventing survey participation or anthropometric data collection were excluded.

### Sampling

We conducted a census in the 10 selected neighborhoods to identify all households with ≥1 eligible adolescent. We drew a random sample of 130 households in each of the 10 neighborhoods: 96 were randomly selected to be surveyed; the remaining 34 households were used as replacements in random order. When multiple eligible adolescents lived in 1 household, 1 adolescent was selected using the random selection function of the computer-assisted personal interview software (Survey Solutions, The World Bank Group).

### Sample size

Assuming a prevalence of nutrient adequacy of 50% (worst-case scenario in terms of sample size), a design effect of 2 (to account for 2-stage sampling, i.e., drawing households from study clusters), 20% of missingness (e.g., refusals, incomplete data, and improbable values), 80% statistical power, and a significance level of 5%, a sample size of 960 households would allow us to detect a difference in prevalence of adequacy of 17 percentage points and an effect size of 0.35 when comparing adolescents in the 5 poorest and the 5 better-off neighborhoods.

### Data collection

Data collection was conducted in person in the adolescent’s mother tongue or in a language the adolescent adequately mastered, among the 4 local languages and English, from September 2021 to October 2021. All data were collected by trained enumerators using electronic questionnaires coded in Survey Solutions and installed on tablet computers. The survey followed a strict COVID-19 protocol involving daily enumerator body temperature monitoring, physical distancing, and face masks for enumerators and respondents.

Enumerators first collected data on household (from the household head) and adolescent (from the adolescent) characteristics at the adolescent’s home. At the end of the interview, they gave the adolescent a standard plastic plate and bowl and invited him/her to use these for eating during the following days and to pay attention to the type and quantity of foods and beverages they consumed. Finally, an appointment was made 2 to 3 d later with one of the highly trained diet enumerators, a team solely responsible for conducting the dietary recalls.

The multiple-pass quantitative 24-h dietary recall surveys were conducted on all days of the week [18]. A second recall, necessary to estimate intra-person variation, was administered in a random subsample of 12% to obtain a minimum of 50 repeat recalls per living-condition stratum (low class or middle class), assuming 15% missing data [19]. This was conducted on a nonconsecutive day within 2 wk of the first recall, generally within 2 to 3 d for operational reasons, and across all days of the week. In pass 1, all foods and beverages consumed during meals and snacks were recorded. In pass 2, detailed information was collected about each food and beverage consumed, such as the cooking method, the place of procurement, and the place of consumption. Respondents were also asked to report on who made the decision leading to the consumption of each food and beverage. In pass 3, quantities consumed were estimated for each food/beverage by weighing an equivalent amount of the food that was consumed, if available, or an equivalent volume of water, raw rice, or playdough, or by using pictures of different sizes of foods, household measures, or price, as relevant, as recommended [18]. Leftovers were estimated using the same approach. Pieces of nutrient-dense meat, fish, or egg included in mixed dishes were quantified separately using playdough models to improve the accuracy of the portion size estimates. For all mixed dishes prepared at home, the unique household recipe was recorded by interviewing the person who cooked them, going through passes 1 to 3 to collect the list and quantities of ingredients. For liquid-based dishes, the final quantity of the cooked dish was estimated by measuring its equivalent volume of water. Finally, in pass 4, the enumerator reviewed all reported foods and beverages with the respondent, who was then asked to recall any additional meals, snacks, or drinks that had not yet been recorded. For mixed dishes obtained outside the home, standard recipes were obtained from a separate survey conducted among food vendors of the study area by the same diet enumerators and from a concurrent study on adolescent diets in Accra [14].

At the end of the interview, adolescents were invited to come to a central location (typically a school) during the weekend. Their weight and height were measured in duplicate by specially trained enumerators. Weight was measured using the UNISCALE (SECA model 874) to a precision of 0.1 kg, and height was measured using a stadiometer (SECA 217) to a precision of 0.1 cm.

### Data management

Energy and protein requirements were calculated using adolescents’ age, sex, weight, and height [20,21]. As recommended, missing weights (*n* = 320) and heights (*n* = 320) were imputed using the age-group- (12‒14 y or 15‒18 y old) and sex-specific sample medians [21]. Energy requirements were calculated for 3 hypothetical levels of physical activity for each individual (sedentary, low active: 30‒60 min of daily moderate activity such as walking, or active: >60 min of these activities).

Conversion factors for densities, household measures, and edible portions were collected through a subsequent survey when not available from the previously mentioned concurrent study [14]. Energy and macronutrient and micronutrient intake were calculated by multiplying food weights by relevant food nutrient composition, obtained from food composition tables and the literature [[22], [23], [24]]. Dietary supplement intake was not considered. A food composition table for the standard recipes was created. We averaged the nutrient composition per 100 g of each recipe across vendors, accounting for micronutrient retention factors from the literature [22] and for measured variation between the weight of the sum of raw ingredients and the weight of the final dish. For standard recipes with discernible pieces of meat, fish, or egg, a version of the recipe without these quantifiable pieces was also created, to be used when the respondent was able to quantify intake of these nutrient-dense foods separately.

### Variable creation

The indicators of interest for this study (study outcomes) included the global diet quality score (GDQS, a measure of overall healthiness of the diet) and several indicators of dietary adequacy: the prevalence of adequate intake (PA) and the mean probability of adequate intake (MPA) of 11 micronutrients, and the PA of fruits and vegetables, fiber, total sugar, proteins, total fats, and saturated fats. We computed the GDQS (range 0‒49) and its additive sub-metrics GDQS healthy (GDQS+; range 0‒32) and GDQS unhealthy (GDQS−; range 0‒17) [25]. GDQS+ is calculated based on the intake of foods from 16 healthy food groups in the past 24 h. A higher score indicates a higher consumption of foods from these healthy food groups. The GDQS− measures the intake in the past 24 h of foods from 7 food groups considered unhealthy. A low consumption or no consumption of unhealthy food groups receives a higher score than consumption above a food group–specific cutoff. For 2 food groups (red meat and whole milk), consumption below a certain amount is considered healthy (score 1 or 2), whereas consumption above a given cutoff point is ranked as unhealthy (score of 0). The total GDQS score increases with a higher consumption of foods from the healthy food groups and a lower consumption of foods from the unhealthy food groups. Findings of a validation study show that the GDQS was positively associated with the healthiness of the diet (based on micronutrient and fiber content) and negatively associated with nutrients that increase NCD risk (added sugar and saturated fat) in adolescents aged 10 to 14 y in 4 countries [26]. GDQS methodology further defines low (GDQS ≥23), moderate (GDQS <23 and ≥15), and high risk (GDQS <15) of poor diet quality. The performance of these cutoffs to approximate nutrient adequacy and NCD risk has not been published [25,27]. Each simple food or ingredient was classified into a GDQS food group. For each standard recipe of a mixed dish, we summed the weight of the raw ingredients by GDQS food group and divided it by the total weight of all raw ingredients to obtain the proportion of each GDQS food group. The amount of each food group consumed from a standard recipe was calculated by multiplying the food group–specific proportion by the total amount of the prepared recipe consumed.

We estimated the distribution of usual intakes of fruits and vegetables, energy, protein, carbohydrates, fiber, total sugar, total fats, saturated fats, cholesterol, and 11 micronutrients using the *ncimultivar* package version 1.0.5 in R (Posit Software, PBC) [28]. This package implements the multivariate method developed by the National Cancer Institute (NCI) for calculating the distribution of usual intake of foods and nutrients from 24-h dietary recalls [29]. We used 200 bootstrap replicates, obtained from random sampling with replacement of 10 neighborhoods (clusters) to account for clustering. Covariates in the Markov Chain Monte Carlo algorithm (200 simulations) were age, sex, stratum (low class or middle class), and whether the recall covered a weekend day.

We calculated the proportion of energy intake from foods/beverages selected independently by the adolescent during the first recall. This was done by summing the energy intake of all foods and beverages consumed in the 24 h for which the adolescent was the sole decision maker and dividing this by their total reported daily energy intake. Similarly, we calculated the proportion of energy intake from foods/beverages obtained at home, at school, and at outlets (defined below). We classified foods and beverages based on where they were prepared and obtained, rather than the origin of the ingredients used to prepare them. For example, raw rice purchased in a store but cooked at home was considered a food obtained at home. The “school” category included the school food court, the school free lunch program (if available), and the school canteen service. The last category (“outlet”) included food vendors (which could have been close to or even on the school premises if they were not part of the school food court), as well as food from “other” sources such as food obtained from churches, sports, social, and other groups, and gifts (which were mainly from friends or relatives). Because these other food sources only represented 1.4% of the total energy intake, we added this category under the “outlets” category. Finally, we calculated the proportion of energy intake from foods/beverages by place of consumption: at home, at school (anywhere on the school premises), or elsewhere.

### Data analysis

Statistical analyses were conducted using Stata 18.0 (StataCorp). We calculated descriptive statistics (frequencies, means, and SE accounting for clustering) using the svyset command in Stata. We presented total energy intake, energy intake by GDQS food group, and energy intake by meal, by food selection decision maker, by place where food/beverage was obtained, and by place of consumption.

For the first objective, which was to describe the healthiness of the diet, we presented descriptive statistics for the GDQS and the adequacy indicators. To compute the latter, we used the *ncimultivar* output datasets of simulated usual intakes described above, which comprise pseudo-persons with the same covariate distribution as the original or bootstrap datasets. We calculated the PA for proteins and all micronutrients through the probability approach [30]; that is, we calculated for each individual a probability of adequate intake using recommended average requirements and related coefficients of variation, using WHO protein requirements [21] and the harmonized nutrient reference values [3] for micronutrients. We assumed a semi-refined diet for zinc and 16% bioavailability for iron because of the high consumption of animal-source foods containing heme iron in the study population. For the skewed distribution of iron requirements, we adjusted the Institute of Medicine (IOM) iron requirements tables [31] using values from the European Food Safety Authority (EFSA) as recommended in harmonized nutrient reference values [3], before applying the probability approach. More specifically, we first recalculated IOM intake cutoffs assuming 16% bioavailability. Next, we added the difference between EFSA and IOM average requirements to each cutoff value of the IOM summary tables for iron. For energy intakes, we calculated for each individual the mean adequacy ratio (AR), defined as the ratio of usual energy intake over estimated average energy requirement, multiplied by 100. Considering energy intake can be inadequate if too low as well as too high, we did not cap the AR at 100. We calculated an AR for each of 3 levels of physical activity to obtain ARs for a sedentary population, a low-active population, or an active population.

We estimated the mean usual intakes, probabilities of adequacy of the 11 micronutrients, and mean AR for energy across all pseudo-persons of the dataset simulated using the observed data. The mean probability of adequate intake of 11 micronutrients was calculated by appending and averaging the PA values of the 11 micronutrients. We calculated the PA for fruits and vegetables, fiber, total sugar, total fats, and saturated fat intake as the proportion of usual intakes meeting the WHO recommendation [[32], [33], [34], [35]]. We applied the recommendation for free sugar (<10% of caloric intake) to total sugar because the food composition table compiled total sugar [23]. We calculated SEs by calculating the variance of each of these estimates across the 200 bootstraps. As instructed, we removed the replicates that did not converge from the calculation of SEs [28]. We also ran this analysis separately by living-condition stratum (low class or middle class). Within each bootstrap, we appended output datasets and used linear regression to model the effect of the class on each indicator of interest, adjusting for age, sex, and weekend day. *P* values were then calculated from bootstrap distributions of coefficients. Statistical inference was based on the t-distribution using appropriate degrees of freedom.

Because the National Cancer Institute method has been updated recently, we confirmed the robustness of our results by estimating the usual intakes and adequacy indicators using the previous method implemented in the Simulating Intake of Micronutrients for Policy Learning and Engagement (SIMPLE) macro in SAS [36] (Supplemental Text 1). The robustness analysis used outputs from the macro, which directly calculates mean usual intakes, probabilities of adequacy, mean AR for energy, and related SEs. For the second objective, which was to assess whether adolescent food selection and where food was obtained were associated with the healthiness of the diet, we first quantified the proportion of energy intake from each healthy and unhealthy food group that came from foods selected independently by adolescents. Food groups were highlighted when this proportion was ≥10% higher than the overall proportion of total energy intake derived from independently selected foods. The same approach was used to assess food group–specific energy intake from foods obtained at home, at school, and from food outlets. We then used mixed-effects regression models with age, sex, and stratum as fixed effects and cluster (neighborhood) as a random effect. Dependent variables were GDQS indicators and reported daily fruit and vegetable, energy, and nutrient intakes. We regressed each of the dependent variables on the proportion of energy intake consumed from foods/beverages selected independently by the adolescent or on the proportion of energy intake obtained at school and obtained at outlets. For foods acquired from outlets, we also assessed whether the place of consumption (at home, at school, or elsewhere) was associated with the indicators of interest. The food and nutrient intake–dependent variables were Box-Cox transformed to normalize the distribution of residuals. All models, except those with GDQS as the dependent variable, included total energy intake as a covariate. Linear regression coefficients were standardized to make them comparable across nutrients. Standardized coefficients represent the average change in SDs of the (Box-Cox transformed) dependent variable associated with a 1 SD change in the independent variable.

To facilitate the interpretation of the magnitude of the association for results with Box-Cox transformed dependent variables, we calculated the predicted values using the estimated regression parameters under the following 2 extreme hypothetical scenarios: *1*) no energy intake was consumed from foods/beverages selected independently, and *2*) all energy intake was consumed from foods/beverages selected independently. Likewise, we calculated the predicted dependent variable under the following 3 different scenarios of where consumed food was obtained: *3*) no energy intake from foods/beverages obtained at school or at outlets; *4*) all energy intake from foods/beverages obtained at school; and *5*) all energy intake from foods/beverages obtained at outlets. We then back-transformed these predicted values using the lambda of the Box-Cox transformation and plotted the relative change when shifting from scenario *1*) to *2*) and when shifting from scenarios *3*) to *4*) and from *3*) to *5*).

### Ethics

The study received ethical clearance from the Noguchi Memorial Institute for Medical Research institutional review board (IRB) (CPN 022/19-20 amendment 2021) and from the International Food Policy Research Institute IRB (PHND-20-0201M). Informed consent to participate in the survey was documented through a signature (or a fingerprint in case of illiteracy) from the parent or legal guardian. We also obtained assent from the adolescents.

## Results

### Characteristics of the sampled adolescents and of their diet

We enrolled 96 households in each cluster, and 1 adolescent per household, of which 25% came from the replacement list (Supplemental Figure 1). Households were typically small, and <1 out of 10 experienced moderate to severe hunger (Table 1). Approximately half of the households had a female head, and 7 out of 10 household heads were literate. Adolescents in our sample were balanced across sex, and the average age was 14 y. More than half attended junior high school, whereas 4 out of 10 were still in primary school. Nearly all adolescents received pocket money, which they mainly used to buy breakfast, lunch, and/or snacks and to pay for after-school classes.

**TABLE 1:** Characteristics of the sample of urban Ghanaian adolescents^1^

	*n*	Proportion (%) or mean ± SE
**Household**	***n* = 960**	
Size, members	—	4.2 ± 0.090
Household hunger^2^	80	8.3
Lives in a wealthier neighborhood	480	50
**Household head**	*n* = 958	
Male	470	49
Ever attended school	816	85
Can read and write	672	70
**Adolescent**	*n* = 960	
Age, y	—	14 ± 0.042
Male	445	46
Attends a public school	578	60
School attended		
Primary	372	39
Junior high school	553	58
Senior high school or higher	35	3.7
Received pocket money in the past week	929	97
Use of pocket money on the previous day for		
Breakfast	694	75
Lunch	643	69
Snacks	283	30
Dinner	12	1.3
Drinks	41	4.4
Paying for extra classes	425	46
Savings	199	21
Walk to school	821	86
Linear distance^3^ to school (one way), m	—	824 ± 47
Belongs to a social group or club at school	195	20
Belongs to a social group or club outside school	105	11
Used to attend a religious service	862	90
	*n* = 591	
BMI-for-age *z*-score^4^	—	−0.11 ± 1.1

1 Values are proportions expressed in percentages (%) or mean ± SE.

2 Household hunger (moderate or severe) defined as household hunger score >1 [37].

3 Calculated using global positioning system coordinates of the house and of the school.

4 This value should be interpreted with caution given the large proportion of missing data, not missing at random.

Diet data were collected in 95% of the sample (*n* = 911; Supplemental Figure 1). The energy intake was, on average, in line with or slightly higher than requirements, assuming a low-active lifestyle for all (Table 2), and 44% and 49% had excessive total fat and sugar intake. Although intake of protein, niacin, vitamin B-12, and iron was adequate for >90% of adolescents, prevalence of adequacy was <10% for fruits and vegetables, riboflavin, and calcium, and <50% for folate, vitamin C, and zinc (Table 2). Results of the robustness analysis were very similar (Supplemental Table 1). Nearly all adolescents were at moderate or high risk of poor diet, based on their GDQS scores (Table 2). There were no significant differences between the low and the middle class of living conditions for any of the indicators of interest (Supplemental Table 2).

**TABLE 2:** Distribution of usual energy and nutrient intakes and of the global diet quality scores, prevalence of nutrient adequacy, mean probability of adequacy, and risk of poor diet in a sample of urban Ghanaian adolescents (*n* = 911)

	Intake				Adequacy, %	
	Mean	SE	P25	P75	Mean	SE
Usual energy and nutrient intake and adequacy indicators						
Energy^1^, kcal	2370	42	1999	2706	112	2.2
Protein,^2^ g	77	2.1	65	88	97	1.3
Carbohydrates, g	329	5.1	282	371	—	—
Fiber,^3^ g	27	0.78	22	31	55	4.5
Sugar,^4^ g	60	1.5	46	72	51	2.5
Total fats,^4^ g	76	2.6	62	87	56	4.0
Saturated fats,^4^ g	21	0.75	17	24	76	3.1
Cholesterol, mg	268	20	199	325	—	—
Vitamin A,^2^*μ*g RAE	757	68	341	947	57	5.7
Thiamin,^2^ mg	0.86	0.020	0.70	0.99	58	2.5
Riboflavin,^2^ mg	0.80	0.031	0.67	0.91	6.7	1.2
Niacin,^2^ mg	16	0.48	13	18	91	3.3
Vitamin B-6,^2^ mg	1.4	0.042	1.1	1.6	61	5.0
Folate,^2^*μ*g	222	4.1	178	258	46	2.4
Vitamin B-12,^2^*μ*g	4.5	0.31	3.4	5.4	99	2.0
Vitamin C,^2^ mg	60	2.1	41	74	35	2.9
Calcium,^2^ mg	346	8.3	293	392	0.00026	0.0038
Iron,^2^ mg	16	0.40	14	19	99	0.6
Zinc,^2^ mg	9.3	0.23	7.8	11	45	3.7
MPA,^5^ %	—	—	—	—	54	1.6
Fruits and vegetables,^3^ g	225	6.2	171	271	2.3	1.2
GDQS indicators^6^						
GDQS+ points^7^	8.1	0.082	5.5	10.5	—	—
GDQS− points^7^	8.7	0.064	8.0	10	—	—
GDQS points^7^	17	0.12	14.25	19.25	—	—
Low risk of poor diet^8^	—	—	—	—	4.4	—
Moderate risk of poor diet^8^	—	—	—	—	64	—
High risk of poor diet^8^	—	—	—	—	32	—

Abbreviations: GDQS, global diet quality score; MPA, mean probability of adequate intake of 11 micronutrients; P25, 25th percentile of the distribution of usual intakes; P75, 75th percentile of the distribution of usual intakes; RAE, retinol activity equivalent; SE, standard error around the mean calculated from the 200 bootstraps.

1 Energy adequacy ratio (AR) calculated as the ratio of energy intake over energy requirement, assuming that all individuals in the sample have a physical activity level defined as low active. Assuming a sedentary population, the energy AR would be 132% ± 2.7%. Assuming an active population, the energy AR would be 97% ± 1.4%.

2 Prevalence of adequate intake calculated with a probability approach considering the distribution of nutrient requirements.

3 Prevalence of adequate intake calculated as the prevalence of usual intake meeting or exceeding the minimum threshold.

4 Prevalence of adequate intake calculated as the prevalence of usual intake below the maximum threshold.

5 Mean probability of adequacy calculated by averaging the prevalence of adequacy across the 11 micronutrients.

6 Data from first recall only.

7 GDQS+ (range 0‒32 points) scores positively the intake of 16 healthy food groups and GDQS− (range 0‒17 points) scores positively the limited intake of 7 unhealthy food groups and 2 food groups unhealthy in excessive amount. GDQS (range 0‒49 points) is the sum of GDQS+ and GDQS−.

8 Value presented is proportion of adolescents with low risk of poor diet defined as GDQS ≥23 points or with moderate risk of poor diet defined as 15 ≤ GDQS <23 points or with high risk of poor diet defined as GDQS <15 points.

**FIGURE 1:**
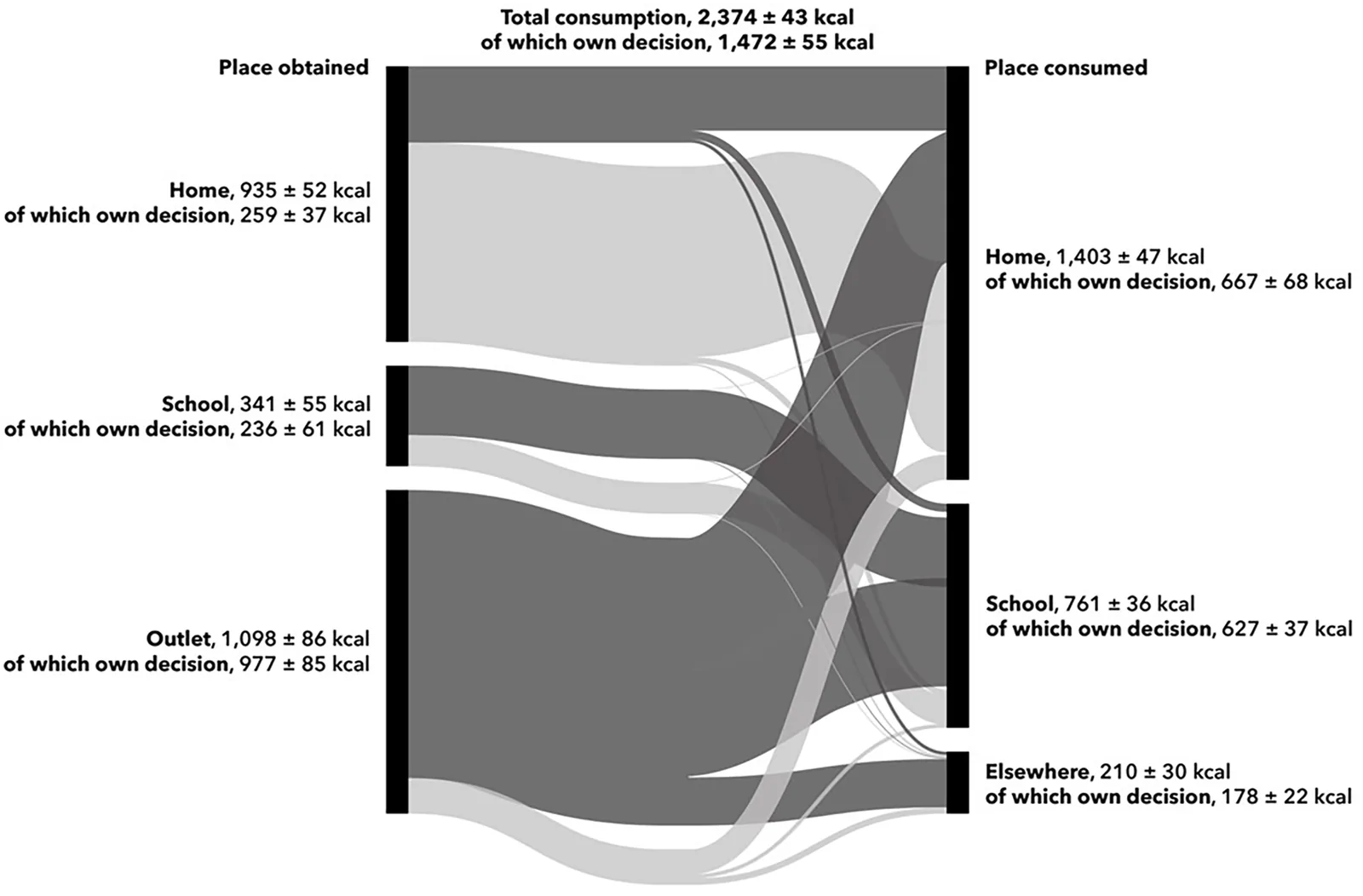
Mean energy intake of adolescents by food selection decision maker, source, and place of consumption (*n* = 911). Dark grey shows energy intake from adolescent-selected foods; light grey shows energy intake from foods selected by someone else, with or without the adolescent.

Foods/beverages obtained at home (on average 39% of total energy intake) were almost exclusively consumed at home (Figure 1). Similarly, foods/beverages obtained at school (14% of energy) were almost exclusively consumed at school. Foods/beverages obtained from outlets represented approximately half of adolescents’ energy intake and were primarily consumed at home (half) and at school (one-third). Adolescents independently selected nearly two-thirds of their energy intake. Independent selection by adolescents was highest for foods/beverages obtained in outlets (89% of energy intake from outlets was independently selected) and at school (69%). On average, 13% of the energy intake was consumed as snacks (297 ± 17 kcal), whereas the remaining energy intake was almost equally divided between breakfast (687 ± 15 kcal, 29% of energy intake), lunch (649 ± 34 kcal, 27%), and dinner (741 ± 28 kcal, 31%). Breakfast was primarily obtained from outlets, lunch from school and outlets, dinner from home, and snacks from outlets (Supplemental Figure 2).

Nearly all adolescents consumed “other vegetables” (vegetables not belonging to the deep orange, green leafy, or cruciferous category; GDQS+ food group), refined grains and baked goods (GDQS− food group), and liquid oils (healthy) in the past 24 h (Table 3). More than half of adolescents reported having consumed fish/shellfish (healthy), sweets (unhealthy), and white roots and tubers (unhealthy). Adolescent-selected food intake was higher by ≥10% in 3 of the 11 healthy food groups and in 4 of the 6 unhealthy food groups consumed by ≥10% of adolescents (Table 3). Likewise, the diets obtained at school or in outlets were proportionally higher in 6 and 4 of these 11 healthy food groups, respectively; and in 3 and 4 of these 6 unhealthy food groups, respectively. In comparison, the diet obtained at home was higher in 6 of 11 healthy and only 1 of 6 unhealthy food groups.

**TABLE 3:** Mean reported daily energy intake of global diet quality score food groups, by food selection decision making and source of food and beverage, in a sample of urban Ghanaian adolescents, first recall (*n* = 911)

	Consumed food group (≥5 g), %	Reported energy intake, kcal		Proportion of reported energy intake selected independently, %^1^	Proportion of reported energy intake, %		
		Mean	SE		From home^1^	From school^1^	From outlets^1^
All foods/beverages	100	2374	43	62	39	14	46
GDQS healthy food groups:							
Other vegetables	97	60	2.5	52	**49∗**	**17**∗	34
Liquid oils	93	355	17.5	62	35	**23**∗	42
Fish and shellfish	70	110	5.4	36	**68**∗	7.8	24
Whole grains	56	115	9.0	61	39	6.6	**54**∗
Eggs	41	45	5.3	**73**∗	24	15	**61**∗
Poultry and game meat	36	122	12	54	**44**∗	**16**∗	40
Deep orange vegetables	34	8.9	0.86	**77**∗	25	**21**∗	**54**∗
Dark green leafy vegetables	26	1.7	0.21	34	**68**∗	13	20
Legumes	25	64	5.8	**74**∗	15	**28**∗	**58**∗
Cruciferous vegetables	24	1.7	0.17	53	**43**∗	**22**∗	35
Nuts and seeds	22	57	4.7	45	**70**∗	3.7	26
*Other fruits*^2^	*4.4*	*8.3*	*1.7*	***74***	*33*	*7.3*	***60***∗
*Citrus fruits*^2^	*3.5*	*3.8*	*0.95*	***80***∗	*38*	*3.7*	***58***∗
*Low-fat dairy*^2^	*2.5*	*1.5*	*0.49*	***82***∗	*1.3*	*1.3*	***97***∗
*Deep orange fruits*^2^	*1.5*	*3.7*	*1.3*	***91***∗	*14*	*7.3*	***78***∗
*Deep orange tubers*^2^	*0*	—	—	—	—	—	—
GDQS unhealthy food groups in excessive amount:							
High-fat dairy	24	21	2.9	**82**∗	**58**∗	8.1	34
Red meat	24	43	5.4	27	**71**∗	7.3	22
GDQS unhealthy food groups:							
Refined grains and baked goods	96	648	35	66	34	**16**∗	50
Sweets and ice cream	67	185	12	**88**∗	21	6.6	**72**∗
White roots and tubers	65	247	22	40	**62**∗	6.7	32
Purchased deep fried foods	53	155	24	**84**∗	0	**24**∗	**76**∗
Processed meat	23	20	2.6	**86**∗	15	**21**∗	**65**∗
Sugar-sweetened beverages	21	36	3.1	**91**∗	14	11	**74**∗
*Juice*^2^	*6.0*	*9*	*1.7*	***88***∗	*19*	*5.1*	***76***∗

Abbreviation: GDQS, global diet quality score.

1 Superscript star indicates that the proportion is ≥1.1 times larger than the overall proportion reported in the first row (all foods/beverages). For instance, 49% of energy from “other vegetables” was consumed at home, which is higher than the proportion of total energy intake from foods consumed at home (39%). This indicates that the home diet is proportionally higher in “other vegetables” than the overall diet.

2 indicates food groups consumed by <10% of adolescents.

### Association between the healthiness of the diet and adolescent food selection

**FIGURE 2:**
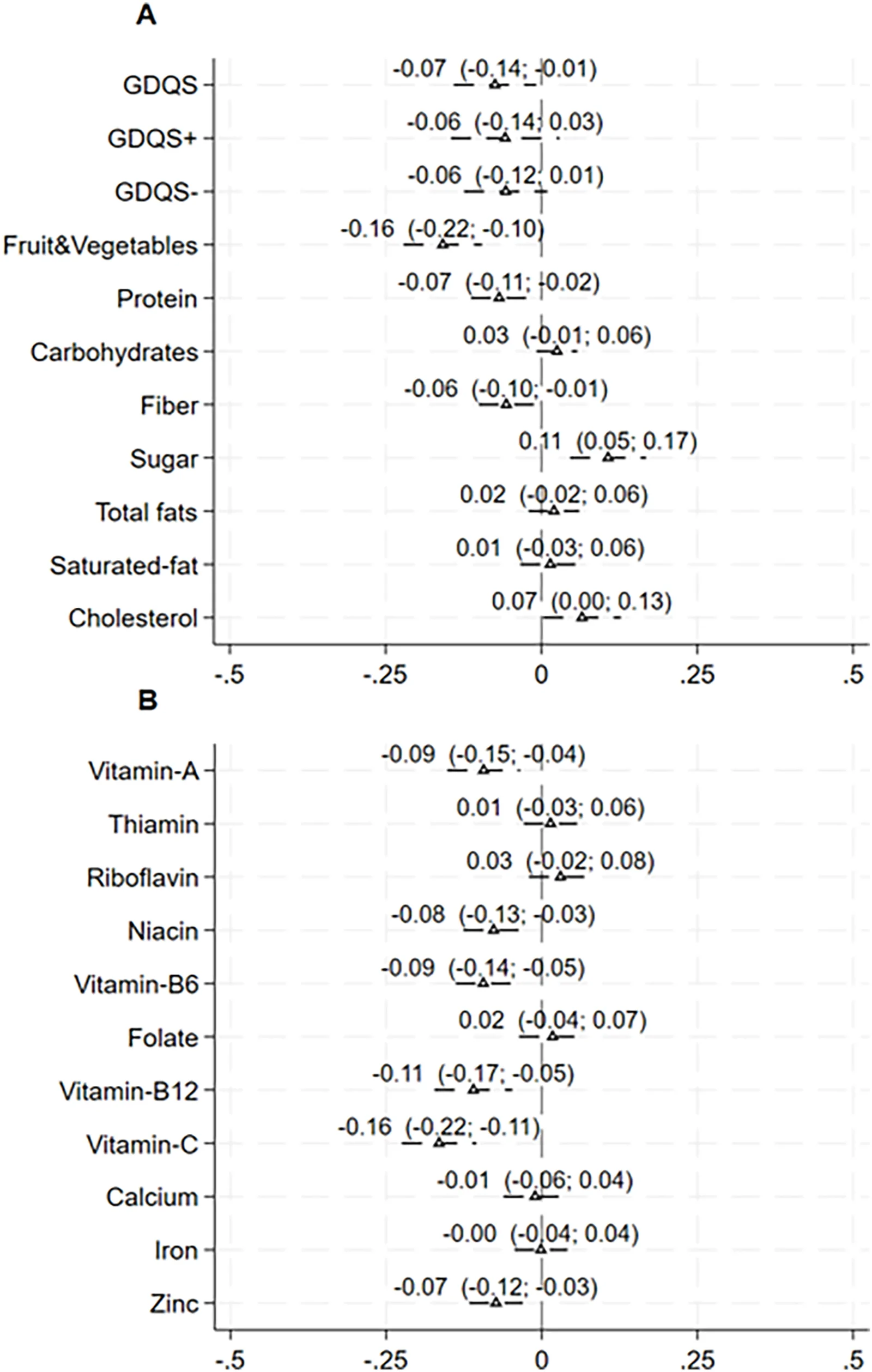
Adjusted standardized linear associations between adolescent-selected food intake and the healthiness of the diet (*n* = 911 in all models). The independent variable for all models is the proportion of energy intake from foods and beverages selected independently by the adolescent. The dependent variables are GDQS indices, reported fruits and vegetables intake, and reported macronutrient intake (A) and reported micronutrient intake (B). X-axes present standardized β coefficients and the standardized 95% confidence intervals in parentheses, which can be interpreted as the change in the (Box-Cox transformed) diet indicator expressed in SDs associated with a 1 SD increase in the proportion of energy intake from adolescent-selected foods. All models used age, sex, and stratum as fixed effects, and neighborhood as a random effect. Except for GDQS indicators, dependent variables were Box-Cox transformed, and models were further adjusted for total energy intake. GDQS, global diet quality score; GDQS−, global diet quality score unhealthy; GDQS+, global diet quality score healthy.

Adolescents who independently selected a larger proportion of their energy intake had diets that were unhealthier (GDQS, *P* < 0.05), were lower in fruit and vegetables, protein, and fiber, and higher in total sugar and cholesterol (all *P* < 0.05; Figure 2A). Furthermore, we found significant negative associations between greater adolescent-selected food intake and the intake of the following 6 of the 11 micronutrients studied: vitamin A, niacin, vitamin B-6, vitamin B-12, vitamin C, and zinc (all *P* < 0.05; Figure 2B).

To assess the magnitude of these associations, we compared the intake of hypothetical adolescents choosing all vs. none of their foods independently. In the case of complete independence, adolescents would consume 34% less fruit and vegetables, 10% less protein, 9% less fiber, 11% to 35% less of 6 micronutrients, 31% more total sugar, and 23% more cholesterol than hypothetical adolescents choosing none of their foods solely by themselves (Supplemental Figure 3). Their GDQS would be 6% lower.

### Association between the healthiness of the diet and the food environment where food was obtained

**FIGURE 3:**
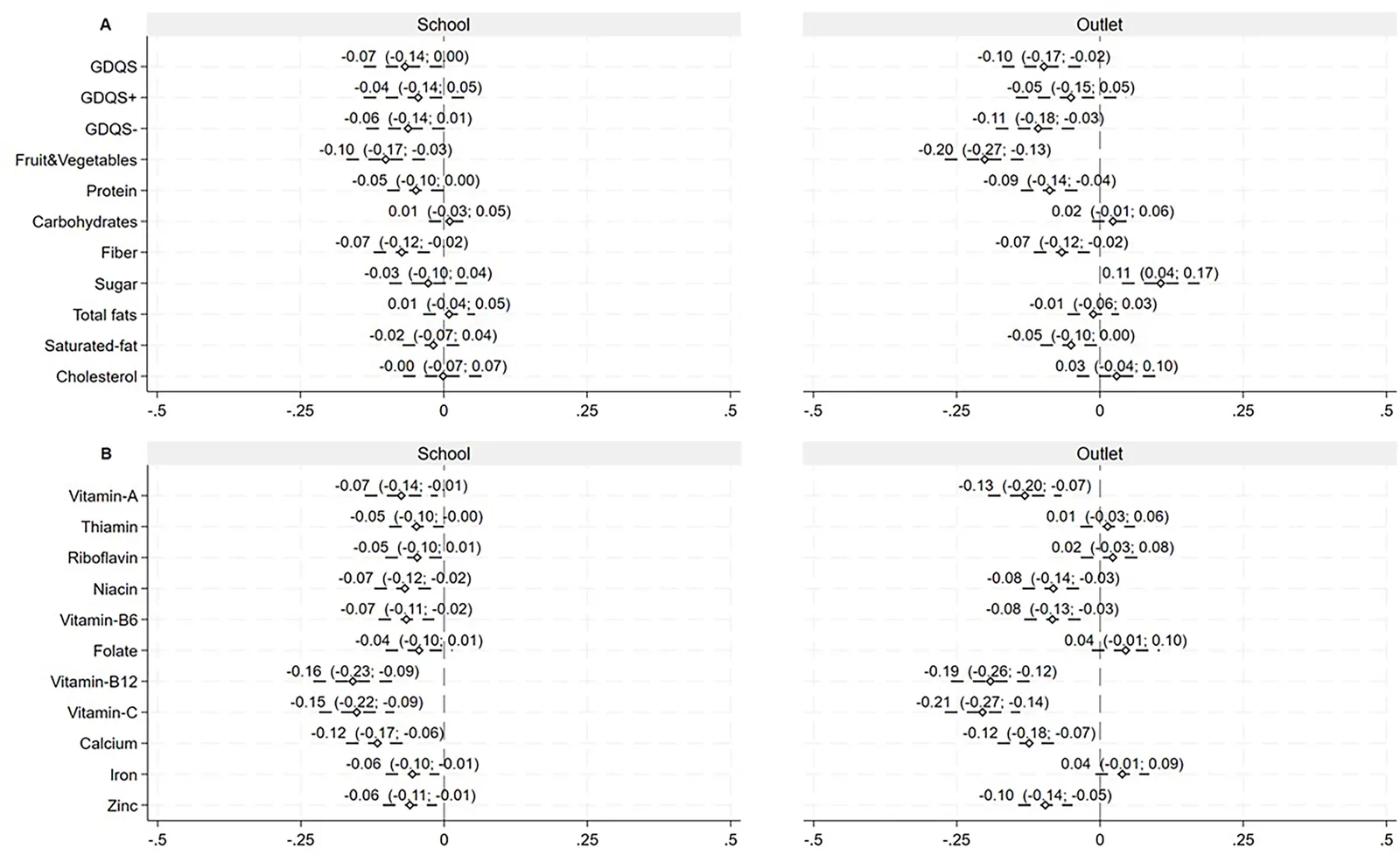
Adjusted standardized linear associations between the external food environment and the healthiness of the diet (*n* = 911 in all models). The independent variable for all models is the proportion of energy intake from foods and beverages obtained directly from the food environment (school or outlets). The dependent variables are GDQS indices, reported fruits and vegetables intake, and reported macronutrient intake (A) and reported micronutrient intake (B). X-axes present standardized β coefficients and the standardized 95% confidence intervals in parentheses, which can be interpreted as the change in the (Box-Cox transformed) diet indicator expressed in SDs associated with a 1 SD increase in the proportion of energy intake from foods obtained from school or from outlets. All models used age, sex, and stratum as fixed effects, and neighborhood as a random effect. Except for GDQS indicators, dependent variables were Box-Cox transformed, and models further adjusted for total energy intake. GDQS, global diet quality score; GDQS−, global diet quality score unhealthy; GDQS+, global diet quality score healthy.

Consuming a larger proportion of energy obtained from school or outlets, respectively, was significantly associated with a lower intake of fruits and vegetables, fiber, and 7 (outlets) or 9 (school) of 11 micronutrients (all *P* < 0.05; Figure 3). Hypothetical adolescents consuming everything from outlets or schools had lower intakes for these foods and nutrients by 10% to 52% than adolescents consuming only food from home (Supplemental Figure 3). In addition, consuming a larger proportion of energy from foods acquired from outlets was associated with lower GDQS and GDQS− scores, lower protein and higher total sugar intake (all *P* < 0.05; Figure 3A). Effects of −6%, −7%, −13%, and +30% for these dependent variables were found when comparing hypothetical adolescents exclusively consuming foods from outlets to adolescents consuming only food from home (Supplemental Figure 3). The results for foods acquired at an outlet were not modified substantively when estimated separately by place where the food was eventually consumed (home, school, or elsewhere; Supplemental Figure 4).

## Discussion

Our sample of adolescents living in low- and middle-class neighborhoods of Accra consumed unhealthy and nutritionally inadequate diets. These adolescents independently selected ≤62% of their total energy intake and purchased 61% of their energy from their food environments, including schools and food outlets. Greater adolescent-selected food intake and a larger proportion of food obtained from these food environments were associated with less healthy diets.

Excessive intake of total sugar and total fats was observed in 49% and 44% of our sample, respectively, consistent with the observed widespread consumption of unhealthy foods. High free sugar and fat intakes increase risk of unhealthy weight gain and, in the case of sugar, risk of dental caries [33,35]. There is evidence that preference for sweet taste may be higher in childhood and decline in adulthood, driven by basic biology [38]. Nevertheless, our results seem to support a nonnegligible preference for sweet taste in this population of adolescents. Moreover, 40% to 93% of adolescents had insufficient intakes of fiber and of vitamin A, thiamin, riboflavin, vitamin B-6, folate, vitamin C, and zinc, and none consumed enough calcium. These nutrient gaps are due to inadequate intake of healthy foods, namely, the insufficient intake of vegetables and the infrequent consumption of most healthy food groups. Our findings align with previous limited evidence from Ghana and other low- and middle-income countries (LMICs) showing the high prevalence of micronutrient inadequacies in this age group, including in urban settings [14,17,39,40]. In contrast, micronutrient adequacy in adolescents from high-income countries is considerably higher, except for calcium [40,41]. Overall, micronutrient deficiencies increase risk of morbidity, cognitive developmental delays, and mortality and reduce educational attainment and economic productivity [[42], [43], [44], [45]]. In adolescent girls, they may increase the odds of future micronutrient deficiencies and associated morbidities, which can lead to adverse birth outcomes and poor anthropometric outcomes in the offspring, contributing to the intergenerational cycle of undernutrition and growth faltering [42,44].

Although adolescent diets were generally unhealthy and inadequate in our sample, they met dietary requirements for protein, iron, vitamin B-12, and niacin, reflecting a high consumption of animal-source foods. These findings are in line with the latest national micronutrient survey that found low levels of vitamin B-12 deficiency in females of reproductive age and of iron deficiency anemia in young children and females of reproductive age in the Southern Belt part of the country where Greater Accra is located [46].

Further to highlighting the main gaps in the diets of adolescents in Accra, our findings add to the limited evidence from LMICs by showing that, in this population, higher adolescent-selected food intake and greater engagement with the external food environment—largely through the purchase of foods using pocket money—are associated with less healthy diets, higher intake of nutrients consumed in excess, and lower intake of foods and nutrients with insufficient intake.

Adolescents in our study had a high level of independence in food selection and obtained most of their energy from the external food environment. This independence was facilitated by the pocket money that nearly all adolescents received from their parents, which they reported using to purchase meals, snacks, and beverages away from home. An observational study in urban Ghana similarly found that complete meals were the most frequently purchased foods at school by students, including adolescents, followed by sweetened drinks, savory snacks, confectionery, and fried foods [10]. Another recent study in Accra reported that greater amounts of pocket money were associated with both higher dietary diversity and greater consumption of unhealthy food groups [47]. Evidence from Ethiopia also shows that adolescents tend to spend their pocket money on fried foods, sweets, and sugary beverages rather than on fruits [48].

Our data do not allow us to disentangle the mechanisms linking adolescent decision making and poorer food selection. Because adolescent-selected food intake in this setting largely overlapped with purchasing food at school or in outlets, adolescent food selection was constrained by the availability and affordability of healthy options in the external food environment. Other studies in urban Ghana have found that adolescent food selection was strongly influenced by perceptions of food safety, affordability, and quality [13,49]. It is possible that unhealthier foods, such as processed and packaged foods, are considered safer and more affordable, hence selected over healthier alternatives. However, empirical evidence on the extent to which these determinants drive adolescents’ selection of unhealthy foods remains limited, beyond known factors such as their palatability. Studies among adolescents in high-income countries attempting to quantify the influence of food environment characteristics, such as the healthiness of food offerings, and the price, availability, promotion, and placement of foods on dietary intake show mixed results, with some indicating links between exposure to unhealthy food outlets and unhealthy food consumption [50]. A review of urban food environments in LMICs, but not specifically on adolescents, showed associations between the availability of certain foods in the home and school food environments and their consumption [51]. However, the review did not present results by healthy or unhealthy foods or clarify the direction of associations with dietary outcomes.

A key challenge in studying these associations is defining and characterizing each individual’s food environment. In our study, we assessed where each food consumed was obtained and found that obtaining a greater proportion of food from home may have protected adolescents from the potentially harmful effects of the external food environment, including the school food environment and outlets, which were both associated with poorer diet. However, we do not have the information to clarify whether this protective effect resulted from the greater healthiness of foods traditionally prepared or stored at home compared with options obtained away from home, or whether it resulted from a deliberate parental effort to provide healthier food and diet options at home.

Key strengths of our study include the relatively large sample size and the detailed assessment of the origin of each food and beverage consumed and who chose it. By focusing on consumed foods rather than the food environment as an entry point, we were able to overcome some of the methodological challenges related to characterizing individual food environments. This approach facilitated our analysis of whether food environments contributed to the unhealthiness of adolescent diets. Collecting information on where adolescents procure their food can easily be added to 24-h dietary recalls and help generate rich information on drivers of food healthiness.

In terms of limitations, we must acknowledge that the 24-h recall method is prone to reporting and desirability bias [52]. However, a concurrent validation study conducted in a similar population found acceptable agreement between 24-h recall and weighed records for several key nutrients [14]. Also, we did not collect data on food fortification [53]. Because vitamin A-fortified edible oil was widely consumed, vitamin A adequacy may have been underestimated, whereas low compliance with wheat flour fortification likely limited its impact [46]. Additionally, sugar adequacy may have been underestimated because recommendations for free sugars were applied to total sugars. At least 7% of total sugar intake was provided by the various fruit and vegetable food groups and may not be free sugar (Supplemental Table 3). However, this is unlikely to affect the conclusion that sugar intake should be reduced because the threshold of <10% of energy intake is conservative, and a harder-to-reach threshold of <5% would provide additional health benefits [35]. We imputed body weight and height of about a third of the sample, which may have added some noise to the estimates of energy and protein requirements used to assess adequacy. Another limitation of the study was our limited ability to test interactions between adolescent-selected food intake and obtaining food from the external food environment because almost all foods from outlets were selected independently by adolescents. Of note, we did not adjust for multiple comparisons because the indicators of interest were not conceptually independent and we transparently reported on all tested hypotheses. Applying multiple-comparison adjustments in this context may unnecessarily increase the risk of type II error and mask meaningful associations for individual outcomes. Finally, our study was limited in its ability to explain why foods purchased by adolescents from the external food environment were less healthy. One possibility is that these environments facilitated poorer food selection by predominantly offering unhealthy options. Alternatively, the prominence of unhealthy foods in these settings may reflect a response from the food sector to strong consumer demand—including from adolescents—for such products.

Interventions to enhance adolescent diets in Ghana should be guided by Ghana’s recently released first food-based dietary guidelines [54], which align closely with the dietary challenges identified in our study. The guidelines provide a practical framework for developing interventions that promote healthier diets across life stages, including recommendations specific to adolescents, such as eating a diverse diet, limiting sweets and sugary drinks, avoiding alcohol, and engaging in regular physical activity. They also include guidance on improving food environments overall and in and around schools [55].

At the policy level, the government has a critical role to play in creating a healthy food environment that is safe for adolescents to navigate. Although Ghana has made progress in developing nutrition-related policies, implementation remains a challenge [9]. National experts highlight the need for legislation regulating the promotion and sale of unhealthy foods and beverages in children’s settings and in print or electronic media, with appropriate enforcement [9]. Front-of-package food labeling [56], taxes on unhealthy foods, and, to some extent, subsidies [57] have been found effective at changing consumption of targeted foods, but their impact on adolescent food choice requires further study. Ongoing advocacy efforts aim to mobilize and support the public sector in developing and implementing such policies in Ghana and other African countries [58]. Schools remain a critical platform for promoting healthy diets during adolescence. Evidence shows that multicomponent programs—combining nutrition education, healthier school food environments and food offerings, and parent engagement—can effectively shift dietary behaviors [[59], [60], [61], [62]]. Consistent with these findings, the 2019 Ghana *National Guidelines for Nutrition Friendly Schools* recommend promoting healthy foods, restricting unhealthy foods, regulating foods sold on school premises, and involving parents and teachers in supporting healthier food choices [63]. Further research is needed in LMICs to identify feasible and effective school-based strategies. Efforts targeting parents can complement school-based strategies. Making healthy foods available at home, limiting unhealthy options, and modeling healthy eating have been associated with healthier adolescent diets [64,65]. Platforms to reach adolescents—such as youth clubs, churches, leisure activities, or digital tools including artificial intelligence-assisted phone applications [14]—should also be explored, especially in contexts like Accra where adolescents frequently decide on food selection and frequently engage with food environments.

Our study in Accra offers a compelling snapshot of adolescent interaction with food environments in a city undergoing a rapid nutritional transition. Adolescents in Accra show a high degree of independence in food selection and engagement with the external food environment, regularly selecting unhealthy foods in these settings. This underscores the urgency of addressing unhealthy food environments early in the developmental and environmental nutrition transition to prevent long-term health consequences.

In Accra, as in other African cities, dietary improvements are unlikely to be achieved through single policies. A more coordinated approach is needed to ensure the implementation and enforcement of bundles of policy options that target adolescents, parents, schools, and the food environment, and that leverage both incentive-based and regulatory mechanisms [58]. Our findings call for such approaches for shifting adolescents’ preferences toward healthier foods and diets and for reshaping food environments in Accra and in similar African cities to make healthy foods more available, accessible, and attractive.

## Author contributions

The authors’ responsibilities were as follows – EB: conceptualization, project administration, methodology, formal analysis, writing – original draft; RRZ: data curation, methodology, formal analysis, writing – review and editing; DO: investigation, writing – review and editing; LH: conceptualization, writing – review and editing; RA: conceptualization, investigation, project administration, writing – review and editing; MTR: funding acquisition, conceptualization, writing – review and editing; JLL: funding acquisition, conceptualization, project administration, supervision, writing – review and editing; and all authors: read and approved the final manuscript.

## Data availability

Data will be made available on the CGIAR’s institutional repository at https://cgspace.cgiar.org.

## Declaration of Generative AI and AI-assisted technologies in the writing process

During the preparation of this work, the authors used Microsoft Copilot (GPT-5) in order to assist with language editing of selected sentences, including improving sentence clarity, conciseness, and grammatical correctness. After using this tool/service, the authors reviewed and edited the content as needed and take full responsibility for the content of the publication.

## Funding

This work was supported by Global Affairs Canada (https://www.international.gc.ca/) grant 52308/5252/0200, the CGIAR Research Initiative on Sustainable Healthy Diets through Food Systems Transformation, and the CGIAR Better Diets and Nutrition Science Program. We would like to thank all funders who supported this research through their contributions to the CGIAR Trust Fund. The funders had no role in study design, data collection, analysis and interpretation, decision to submit for publication, or preparation of the manuscript.

## Conflict of interest

The authors report no conflicts of interest.
