# Supplementary material for: Adolescent-Selected Food Intake and Engagement with the External Food Environment Contribute to Unhealthy Diets in Accra, Ghana
Source: Curr Dev Nutr. 2026 Jul 10;10(9):109435. doi: 10.1016/j.cdnut.2026.109435 (PMC13520089; doi:10.1016/j.cdnut.2026.109435)
Supplement: Supplementary file 1 [file mmc1.docx]

**Supplemental material**

**Adolescent-selected food intake and engagement with the external food environment contribute to unhealthy diets in Accra, Ghana**

Becquey, E. et al.

**Supplemental Figure 1: Flow-chart of study participants**

**Supplemental Text 1**

Because the National Cancer Institute method has been updated recently, we confirmed the robustness of our results by estimating the usual intakes and adequacy indicators using the previous method implemented in the SIMPLE macro in SAS (Cary, North Carolina, USA) [1]. This macro is based on the MIXTRAN and DISTRIB macros that were developed by the National Cancer Institute for analysis of non-episodically consumed foods and nutrients[2] and were recommended until the *ncimultivar* package became available [3]. We used the same 200 bootstrap replicates and the same food and nutrient requirements as in the main analysis, and covariates in the MIXTRAN macro were the same as used in the Markov Chain Monte Carlo algorithm. If the MIXTRAN bootstrap output for a nutrient showed a within-to-between person variance ratio higher than 10 (deemed inaccurate [4]), then the second day of intake of the most influential observation for this nutrient was dropped from the calculation until a ratio below 10 was obtained. We used the SIMPLE-Iron macro variation to calculate the PA of iron through the full probability approach [5]. The PA for fruits and vegetables, fiber, sugar, total fats, and saturated fat intake was calculated as the proportion of the population with a usual intake meeting the requirement using the Estimated Average Requirement cut-point method [5] implemented in SIMPLE. We enhanced the SIMPLE macro to calculate the PA for proteins and the 10 remaining micronutrients through the full probability approach instead of the Estimated Average Requirement cut-point approximation [5]. Finally, we adapted the SIMPLE macro to calculate the mean adequacy ratio (AR) for energy, defined as the ratio of usual energy intake over estimated average energy requirement, multiplied by 100. For the first objective, which was to describe the healthiness of the diet, the robustness analysis used outputs from the SIMPLE macro which directly calculates mean usual intakes, probabilities of adequacy, mean adequacy ratio for energy, and related standard errors.

*References*

1	Luo H, Dodd KW, Arnold CD, *et al.* Introduction to the SIMPLE Macro, a Tool to Increase the Accessibility of 24-Hour Dietary Recall Analysis and Modeling. *Journal of Nutrition*. 2021;151:1329–40. doi: 10.1093/jn/nxaa440

2	Tooze JA, Kipnis V, Buckman DW, *et al.* A mixed-effects model approach for estimating the distribution of usual intake of nutrients: the NCI method. *Stat Med*. 2010;29:2857–68. doi: 10.1002/SIM.4063

3	National Cancer Institute. Measurement Error: Impact on Nutrition Research and Adjustment for its Effects. 2025. https://prevention.cancer.gov/tools-resources/measurement-error-impact-nutrition-research-and-adjustment-its-effects#_section_3._software_for_measurement_error_in_nutrition_research_ (accessed 13 October 2025)

4	Davis KA, Gonzalez A, Loukine L, *et al.* Early experience analyzing dietary intake data from the canadian community health survey—nutrition using the national cancer institute (NCI) method. *Nutrients*. 2019;11. doi: 10.3390/nu11081908

5	Institute of Medicine. Using the Estimated Average Requirement for Nutrient Assessment of Groups. *Dietary Reference Intakes, Applications in Dietary Assessment : a report of the Subcommittees on Interpretation and Uses of Dietary Reference Intakes and Upper Reference Levels of Nutrients, and the Standing Committee on the Scientific Evaluation of Diet*. Washington, DC: National Academy of Sciences 2000:73–105.

**Supplemental Table 1: Usual intake and indicators of adequacy of the diet, in a sample of urban Ghanaian adolescents, in the robustness analysis using the SIMPLE macro^1^ (N=911)**

|  | Intake | | | | Adequacy, % | |
| --- | --- | --- | --- | --- | --- | --- |
|  | mean | SE | P25 | P75 | Mean | SE |
| Energy^3^, kcal | 2,373 | 42 | 2,026 | 2,683 | 112 | 2.2 |
| Protein^4^, g | 77 | 2.1 | 65 | 88 | 97 | 1.4 |
| Carbohydrates, g | 329 | 5.0 | 287 | 367 | NA |  |
| Fiber^2^, g | 27 | 0.76 | 21 | 31 | 55 | 4.6 |
| Sugar^5^, g | 60 | 1.6 | 47 | 71 | 51 | 4.0 |
| Total fats^5^, g | 75 | 2.5 | 62 | 86 | 57 | 6.8 |
| Saturated fats^5^, g | 21 | 0.74 | 18 | 23 | 80 | 6.9 |
| Cholesterol, mg | 267 | 17 | 195 | 326 | NA |  |
| Vitamin A^4^, µg RAE | 733 | 74 | 345 | 922 | 57 | 7.0 |
| Thiamin^4^, mg | 0.86 | 0.020 | 0.71 | 0.98 | 59 | 2.5 |
| Riboflavin^4^, mg | 0.80 | 0.030 | 0.66 | 0.91 | 7.0 | 2.0 |
| Niacin^4^, mg | 16 | 0.48 | 13 | 18 | 93 | 4.7 |
| Vitamin B-6^4^, mg | 1.4 | 0.044 | 1.1 | 1.6 | 60 | 5.5 |
| Folate^4^, µg | 221 | 4.4 | 181 | 255 | 46 | 2.6 |
| Vitamin B-12^4^, µg | 4.4 | 0.30 | 3.3 | 5.3 | 99 | 2.4 |
| Vitamin C^4^, mg | 59 | 2.2 | 40 | 74 | 35 | 2.9 |
| Calcium^4^, mg | 346 | 8.8 | 286 | 396 | 0.000 | 0.020 |
| Iron^4^, mg | 16 | 0.45 | 14 | 19 | 100 | 0.50 |
| Zinc^4^, mg | 9.3 | 0.24 | 8.0 | 10 | 44 | 3.6 |
| MPA^6^, % |  |  |  |  | 55 | 1.7 |
| Fruits and vegetables^2^, g | 226 | 6.1 | 176 | 267 | 1.6 | 1.8 |

^1^ Abbreviations used: P25 25^th^ percentile of the distribution of usual intakes, P75 75^th^ percentile of the distribution of usual intakes, MPA mean probability of adequacy, NA not applicable, SE Standard error around the mean calculated from the 200 bootstraps

^2^ Prevalence of adequate intake calculated as the prevalence of usual intake meeting or exceeding the minimum threshold

^3^ Energy adequacy ratio (AR) calculated as the ratio of energy intake over energy requirement, assuming that all individuals in the sample have a physical activity level defined as low active. Assuming a sedentary population, the energy AR would be 132 ± 2.7 %. Assuming an active population, the energy AR would be 97 ± 1.4 %

^4^ Prevalence of adequate intake calculated with a probability approach considering the distribution of nutrient requirements

^5^ Prevalence of adequate intake calculated as the prevalence of usual intake below the maximum threshold

^6^ Mean probability of adequacy calculated by averaging the prevalence of adequacy across the 11 micronutrient

**Supplemental Table 2: Distribution of usual energy and nutrient intakes and of the Global Diet Quality Scores, prevalence of nutrient adequacy, mean probability of adequacy and risk of poor diet in a sample of urban Ghanaian adolescents, by class of living condition (middle class or low class)^1^**

|  | Low class (N=458) | | | | | | | Middle class (N=453) | | | | | | |
| --- | --- | --- | --- | --- | --- | --- | --- | --- | --- | --- | --- | --- | --- | --- |
|  | Intake | | | | | Adequacy, % | | Intake | | | | | Adequacy, % | |
|  | Mean* | SE | P25 | P75 | Mean* | | SE | Mean* | SE | P25 | P75 | Mean* | | SE |
| **Usual intake and adequacy** |  |  |  |  |  | |  |  |  |  |  |  | |  |
| Energy^3^, kcal | 2355 | 49 | 2023 | 2656 | 112 | | 3.2 | 2396 | 84 | 2044 | 2713 | 111 | | 3.8 |
| Protein^4^, g | 76 | 2.4 | 62 | 89 | 97 | | 1.3 | 78 | 4.4 | 66 | 89 | 97 | | 2.8 |
| Carbohydrates, g | 329 | 9.3 | 286 | 368 |  | |  | 330 | 7.7 | 276 | 377 |  | |  |
| Fiber^2^, g | 26 | 1.3 | 21 | 31 | 52 | | 7.5 | 27 | 1.0 | 22 | 32 | 58 | | 5.9 |
| Sugar^5^, g | 59 | 2.5 | 48 | 69 | 51 | | 5.5 | 61 | 3.0 | 43 | 75 | 52 | | 7.2 |
| Total fats^5^, g | 75 | 3.3 | 60 | 87 | 58 | | 3.7 | 77 | 4.9 | 61 | 89 | 57 | | 5.1 |
| Saturated fats^5^, g | 21 | 1.1 | 17 | 24 | 79 | | 3.1 | 21 | 1.4 | 16 | 25 | 77 | | 4.2 |
| Cholesterol, mg | 255 | 29 | 162 | 326 |  | |  | 284 | 31 | 217 | 339 |  | |  |
| Vitamin A^4^, µg RAE | 693 | 66 | 284 | 811 | 48 | | 8.2 | 841 | 160 | 458 | 1047 | 70 | | 8.9 |
| Thiamin^4^, mg | 0.9 | 0.03 | 0.71 | 1.0 | 60 | | 4.8 | 0.86 | 0.03 | 0.71 | 0.99 | 58 | | 3.5 |
| Riboflavin^4^, mg | 0.8 | 0.03 | 0.64 | 0.90 | 7.0 | | 2.3 | 0.82 | 0.07 | 0.70 | 0.93 | 8 | | 3.2 |
| Niacin^4^, mg | 15 | 0.30 | 12 | 18 | 88 | | 5.5 | 16 | 1.1 | 13 | 19 | 92 | | 5.4 |
| Vitamin B-6^4^, mg | 1.4 | 0.05 | 1.1 | 1.6 | 60 | | 5.8 | 1.4 | 0.09 | 1.1 | 1.7 | 61 | | 8.8 |
| Folate^4^, µg | 218 | 5.6 | 176 | 252 | 44 | | 3.4 | 225 | 8.5 | 183 | 260 | 48 | | 4.3 |
| Vitamin B-12^4^, µg | 4.4 | 0.6 | 3.3 | 5.3 | 99 | | 5.3 | 4.5 | 0.52 | 3.4 | 5.3 | 99 | | 3.7 |
| Vitamin C^4^, mg | 60 | 4.5 | 43 | 73 | 36 | | 6.4 | 60 | 2.5 | 39 | 74 | 35 | | 3.0 |
| Calcium^4^, mg | 344 | 10 | 298 | 384 | 0.00 | | 0.00 | 349 | 17 | 289 | 400 | 0.00 | | 0.2 |
| Iron^4^, mg | 16 | 0.48 | 14 | 18 | 99 | | 1.7 | 17 | 0.78 | 14 | 19 | 98 | | 1.6 |
| Zinc^4^, mg | 9.2 | 0.21 | 7.7 | 10 | 43 | | 3.9 | 9.5 | 0.52 | 7.9 | 11 | 46 | | 7.2 |
| MPA^6^, % |  |  |  |  | 53 | | 2.2 |  |  |  |  | 56 | | 3.7 |
| Fruits and vegetables^2^, g | 227 | 13 | 172 | 274 | 2.6 | | 1.8 | 224 | 3.5 | 162 | 275 | 4 | | 1.9 |
| **GDQS indicators^7^** |  |  |  |  |  | |  |  |  |  |  |  | |  |
| GDQS+, points | 8.0 | 0.13 | 5.5 | 11 |  | |  | 8.1 | 0.11 | 6.0 | 11 |  | |  |
| GDQS-, points^8^ | 8.8 | 0.09 | 8.0 | 10 |  | |  | 8.6 | 0.05 | 7.0 | 10 |  | |  |
| GDQS, points^8^ | 17 | 0.21 | 14 | 20 |  | |  | 17 | 0.16 | 14 | 19 |  | |  |
| Low risk of poor diet^9^ |  |  |  |  | 5.5 | |  |  |  |  |  | 3.3 | |  |
| Moderate risk of poor diet^9^ |  |  |  |  | 65 | |  |  |  |  |  | 62 | |  |
| High risk of poor diet^9^ |  |  |  |  | 30 | |  |  |  |  |  | 34 | |  |

^1^ Abbreviations used: GDQS Global Diet Quality Score, MPA mean probability of adequacy, NA not applicable, P25 25th percentile of the distribution of usual intakes, P75 75th percentile of the distribution of usual intakes, SE Standard error around the mean calculated from the 200 bootstraps.

*All P-values >0.05 for the comparison between classes of living condition of mean usual intake indicators, mean GDQS, and mean adequacy indicators for all indicators of interest. P-values were calculated from bootstrap distributions of linear regression coefficients estimating the effect of class on each indicator of interest, adjusting for age, sex, and weekend day. Statistical inference was based on the t-distribution using appropriate degrees of freedom.

^2^ Prevalence of adequate intake calculated as the prevalence of usual intake meeting or exceeding the minimum threshold

^3^ Energy adequacy ratio (AR) calculated as the ratio of energy intake over energy requirement, assuming that all individuals in the sample have a physical activity level defined as low active. Assuming a sedentary population, the energy AR would be 132 ± 2.7 %. Assuming an active population, the energy AR would be 97 ± 1.4 %

^4^ Prevalence of adequate intake calculated with a probability approach considering the distribution of nutrient requirements

^5^ Prevalence of adequate intake calculated as the prevalence of usual intake below the maximum threshold

^6^ Mean probability of adequacy calculated by averaging the prevalence of adequacy across the 11 micronutrients

^7^ Data from first recall only.

^8^ GDQS+ (range 0-32 points) scores positively the intake of 16 healthy food groups and GDQS- (range 0-17 points) scores positively the limited intake of 7 unhealthy food groups and 2 food groups unhealthy in excessive amount. GDQS (range 0-49 points) is the sum of GDQS+ and GDQS-

^9^ Value presented is proportion of adolescents with low risk of poor diet defined as GDQS ≥23 points or with moderate risk of poor diet defined as 15 ≤GDQS <23 points or with high risk of poor diet defined as GDQS <15 points

energy intake AS SNACKS (kcal)

|  | Decided alone | Did not decide alone |
| --- | --- | --- |
| Obtained at home cons. home | 35.47 | 44.26 |
| Obtained at home cons. school | 0.75 | 3.58 |
| Obtained at home else- where | 0.78 | 2.61 |
| Obtained at school cons. home | 0.03 | 1.14 |
| Obtained at school cons. school | 29.8 | 5.63 |
| Obtained at school else- where | 0.14 | 0.16 |
| Obtained in outlets cons. home | 66.51 | 7.91 |
| Obtained in outlets cons. school | 47.66 | 1.94 |
| Obtained in outlets else- where | 46.37 | 2.15 |

energy intake FOR DINNER (kcal)

|  | Decided alone | Did not decide alone |
| --- | --- | --- |
| Obtained at home conso home | 88.64 | 411.69 |
| Obtained at home conso school | 1.85 | 2.78 |
| Obtained at home conso outside | 1.67 | 0 |
| Obtained in canteen conso home | 0.37 | 0.21 |
| Obtained in canteen conso school | 0 | 0 |
| Obtained in canteen conso outside | 0 | 0.07 |
| Obtained in outlet/other conso home | 168.15 | 31.44 |
| Obtained in outlet/other conso school | 2.31 | 0 |
| Obtained in outlet/other conso outside | 26.04 | 5.82 |

energy intake FOR LUNCH (kcal)

|  | Decided alone | Did not decide alone |
| --- | --- | --- |
| Obtained at home conso home | 33.69 | 115.06 |
| Obtained at home conso school | 4.58 | 4.67 |
| Obtained at home conso outside | 3.73 | 4.88 |
| Obtained in canteen conso home | 0.23 | 0.25 |
| Obtained in canteen conso school | 117.55 | 87.58 |
| Obtained in canteen conso outside | 0.62 | 2.73 |
| Obtained in outlet/other conso home | 81.16 | 16.05 |
| Obtained in outlet/other conso school | 126.55 | 7.28 |
| Obtained in outlet/other conso outside | 37.28 | 5.57 |

energy intake FOR BREAKFAST (kcal)

|  | Decided alone | Did not decide alone |
| --- | --- | --- |
| Obtained at home conso home | 60.78 | 76.01 |
| Obtained at home conso school | 21.02 | 10.34 |
| Obtained at home conso outside | 5.92 | 0.46 |
| Obtained in canteen conso home | 1.72 | 1.81 |
| Obtained in canteen conso school | 83.65 | 5.88 |
| Obtained in canteen conso outside | 1.56 | 0 |
| Obtained in outlet/other conso home | 130.69 | 29.93 |
| Obtained in outlet/other conso school | 191.15 | 4.16 |
| Obtained in outlet/other conso outside | 53.79 | 8.05 |

**Supplemental Figure 2: Mean energy intake of adolescents by food selection decision-maker, source and place of consumption, by meal.** N=911. Abbreviation used: cons., consumed. Thick black border with white fill, energy intake from adolescent-selected foods; light grey fill, energy intake from foods selected by someone else, with or without the adolescent. Y-axis were capped at 300 kcal, labels indicate energy intake in kcal and were omitted for categories < 20 kcal.

*

*

*

*

*

*

*

*

*

*

*

*

*

*

*

*

*

*

*

*

*

*

*

*

*

*

*

*

*

*

*

*

*

*

*

*

From independent decision

Predicted change (%) in GDQS and nutrient intake assuming all foods, compared to none, were obtained :

|  | From autonomous decision | At school | At outlets |
| --- | --- | --- | --- |
| GDQS | -4.47451% | -5.8172% | -5.73714% |
| GDQS+ | -5.25797% | -5.84498% | -4.52359% |
| GDQS- | -3.75606% | -5.79173% | -6.84818% |
| Fruit&Vegetables | -34.3537% | -31.7708% | -41.0851% |
| Protein | -10.4786% | -10.7661% | -13.1418% |
| Carbohydrates | 3.47072% | 2.05314% | 3.07557% |
| Fiber | -9.09384% | -16.4517% | -10.4851% |
| Sugar | 31.3451% | -9.89681% | 29.7978% |
| Total fats | 3.78432% | 2.49858% | -2.09735% |
| Saturated fats | 3.03617% | -5.38964% | -9.88051% |
| Cholesterol | 22.9664% | -0.561247% | 9.45795% |
| Vitamin-A | -32.5487% | -36.7149% | -42.0431% |
| Thiamin | 2.23313% | -10.4472% | 2.04376% |
| Riboflavin | 5.35094% | -11.2248% | 3.87618% |
| Niacin | -13.5554% | -17.0068% | -14.0139% |
| Vitamin-B6 | -15.1146% | -15.3122% | -13.3147% |
| Folate | 3.09707% | -10.6303% | 8.00196% |
| Vitamin-B12 | -29.6539% | -52.2011% | -45.146% |
| Vitamin-C | -34.8377% | -43.5945% | -40.7424% |
| Calcium | -1.85254% | -25.3524% | -19.0148% |
| Iron | -0.210795% | -11.9838% | 6.13183% |
| Zinc | -10.9106% | -12.6536% | -13.7458% |

**Supplemental Figure 3:** **Maximum relative predicted difference in GDQS indices and intake of fruit and vegetables, macronutrients and micronutrients, when all versus none of the food was selected independently (using model presented in figure 2) or was obtained at school or at outlets (using model presented in figure 3).** N=911. For box-cox transformed outcomes (all except GDQS indicators), lambda was used to back-transform the predicted outcome. Stars show models for which the outcome was significantly associated with the proportion of energy intake from foods selected by the adolescent or from foods obtained at school or at outlet.

**Supplemental Figure 4: Adjusted standardized linear associations between the proportion of energy intake from foods and beverages obtained in outlets for consumption at home, at school or elsewhere, and healthiness of the diet indicators: GDQS indices, reported fruits and vegetables and reported macronutrient intake (panel A) and reported micronutrient intake (panel B).** N=911 in all models. Values presented in parenthesis are the standardized 95% confidence intervals of the beta coefficient, which can be interpreted as the change in the (box-cox transformed) outcome expressed in standard deviations associated with a one standard deviation increase in the proportion of energy intake obtained in outlets for consumption at home, at school or elsewhere. All models used age, sex, stratum, and proportion of energy intake acquired at school as fixed effects, neighborhood as random effect. Except for GDQS indicators, dependent variables were box-cox transformed, and models further adjusted for total energy intake.

**Supplemental Table 3: Distribution by GDQS food group of the sum of reported total sugar intake across all recalls (N=1,016)^1^**

|  | Proportion of total sugar intake, % |
| --- | --- |
| GDQS healthy food groups: |  |
| Other vegetables | 6.9 |
| Whole grains | 5.4 |
| Other fruits | 1.9 |
| Citrus fruits | 1.4 |
| Deep orange fruits | 0.77 |
| Deep orange vegetables | 0.54 |
| Low-fat dairy | 0.39 |
| Nuts and seeds | 0.31 |
| Legumes | 0.30 |
| Cruciferous vegetables | 0.24 |
| Eggs | 0.12 |
| Dark green leafy vegetables | 0.0 |
| Fish and shellfish | 0.0 |
| Liquid oils | 0.0 |
| Poultry and game meat | 0.0 |
| Deep orange tubers | 0.0 |
| GDQS unhealthy food groups in excessive amount: |  |
| High-fat dairy | 2.9 |
| Red meat | 0.0 |
| GDQS unhealthy food groups: |  |
| Sweets and ice cream | 36 |
| Sugar-sweetened beverages | 16 |
| Refined grains and baked goods | 13 |
| White roots and tubers | 6.3 |
| Purchased deep fried foods | 4.1 |
| Juice*^2^* | 3.6 |
| Processed meat | 0.07 |

^1^Abbreviations used: GDQS Global Diet Quality Score
